# Metabolite Alterations and Interactions with Microbiota in Helicobacter pylori-Associated Gastric Lesions

**DOI:** 10.1128/spectrum.05347-22

**Published:** 2023-06-26

**Authors:** Lei Peng, Yang Guo, Markus Gerhard, Juan-Juan Gao, Zong-Chao Liu, Raquel Mejías-Luque, Lian Zhang, Michael Vieth, Jun-Ling Ma, Wei-Dong Liu, Zhe-Xuan Li, Tong Zhou, Wen-Qing Li, Wei-Cheng You, Yang Zhang, Kai-Feng Pan

**Affiliations:** a Key Laboratory of Carcinogenesis and Translational Research (Ministry of Education/Beijing), Department of Cancer Epidemiology, Peking University Cancer Hospital & Institute, Beijing, China; b PYLOTUM Key Joint Laboratory for Upper GI Cancer, Technische Universität München, Munich, Germany, and Peking University Cancer Hospital & Institute, Beijing, China; c Institute for Medical Microbiology, Immunology and Hygiene, Technische Universität München, Munich, Germany; d German Center for Infection Research, Partner Site Munich, Munich, Germany; e Institute of Pathology, Klinikum Bayreuth, Bayreuth, Germany; f Linqu Public Health Bureau, Linqu, Shandong, China; Jilin University

**Keywords:** *Helicobacter pylori*, gastric metabolites, gastric microbiota, interactions, precancerous lesions

## Abstract

Metabolites and their interactions with microbiota may be involved in Helicobacter pylori-associated gastric lesion development. This study aimed to explore metabolite alterations upon H. pylori eradication and possible roles of microbiota-metabolite interactions in progression of precancerous lesions. Targeted metabolomics assays and 16S rRNA gene sequencing were conducted to investigate metabolic and microbial alterations of paired gastric biopsy specimens in 58 subjects with successful and 57 subjects with failed anti-H. pylori treatment. Integrative analyses were performed by combining the metabolomics and microbiome profiles from the same intervention participants. A total of 81 metabolites were significantly altered after successful eradication compared to failed treatment, including acylcarnitines, ceramides, triacylglycerol, cholesterol esters, fatty acid, sphingolipids, glycerophospholipids, and glycosylceramides, with *P* values of <0.05 for all. The differential metabolites showed significant correlations with microbiota in baseline biopsy specimens, such as negative correlations between *Helicobacter* and glycerophospholipids, glycosylceramide, and triacylglycerol (*P < *0.05 for all), which were altered by eradication. The characteristic negative correlations between glycosylceramides and *Fusobacterium*, Streptococcus, and *Gemella* in H. pylori-positive baseline biopsy specimens were further noticed in active gastritis and intestinal metaplasia (*P < *0.05 for all). A panel including differential metabolites, genera, and their interactions may help to discriminate high-risk subjects who progressed from mild to advanced precancerous lesions in short-term and long-term follow-up periods with areas under the curve (AUC) of 0.914 and 0.801, respectively. Therefore, our findings provide new insights into the metabolites and microbiota interactions in H. pylori-associated gastric lesion progression.

**IMPORTANCE** In this study, a panel was established including differential metabolites, genera, and their interactions, which may help to discriminate high-risk subjects for progression from mild lesions to advanced precancerous lesions in short-term and long-term follow-up.

## INTRODUCTION

Helicobacter pylori is an important risk factor for various gastric disorders, including chronic gastritis, glandular atrophy, intestinal metaplasia (IM), epithelial dysplasia, and even gastric cancer (GC) (1). Eradication treatment has shown great benefits for GC prevention and regression of precancerous lesions (2–4). Pathogenic mechanisms of gastric carcinogenesis involve complex interactions among H. pylori, other gastric microbiota, and associated metabolites, although systematic studies are still needed.

The advances in sequencing technology have revealed altered microbial diversity and different bacterial interactions in GC and precancerous lesions (5, 6). Our previous intervention study confirmed that H. pylori may induce gastric microbial dysbiosis, which can be restored by successful eradication (7). Our subsequent follow-up study further showed that the differential bacteria in the progression-to-dysplasia/GC subjects were enriched in protein and adipose metabolism pathways by microbial functional-capacity prediction (8). However, the exact microbiota-metabolite interactions still need integrative microbiome and metabolomics confirmation.

Recent studies have reported that interplays between the gut microbiota and metabolites may regulate inflammation and the immune system in colorectal carcinogenesis, such as between *Bifidobacterium*, *Lactobacillus*, and short-chain fatty acids (9–11). An integrative microbiome and metabolomics study in GC tissues found significant correlations between *Helicobacter*, *Lactobacillus*, and differential metabolites (12). Nevertheless, the microbiota-metabolite interactions in the early stage of gastric lesion progression during H. pylori infection, which are important for a deeper understanding of GC etiology and prevention, still remain unclear.

In the present study, we compared the metabolomics profiles of paired gastric biopsy specimens at baseline and follow-up time points from subjects with successful or failed anti-H. pylori treatment based on a prospective population-based cohort. The differential metabolites by H. pylori eradication were integrated with the differential microbiota in the same intervention subjects. This study provided us a unique opportunity to unravel the possible interactions between the gastric microbiota and metabolites in the early stage of precancerous lesion progression.

## RESULTS

The baseline characteristics of successful eradication and failed treatment groups are presented in Table S1 in the supplemental material. No significant differences in baseline age, delta-over-baseline (DOB) value in [^13^C]urea breath test (^13^C-UBT), body mass index (BMI), smoking habits, alcohol consumption, or presence of active gastritis or gastric lesions were found between successful H. pylori eradication and failed treatment groups (*P* > 0.05 for all). There was a higher frequency of male subjects in the failed treatment group than in the successful eradication group (70.2% versus 51.7%; *P = *0.043).

### Alterations of gastric metabolites by H. pylori eradication.

**FIG 1:**
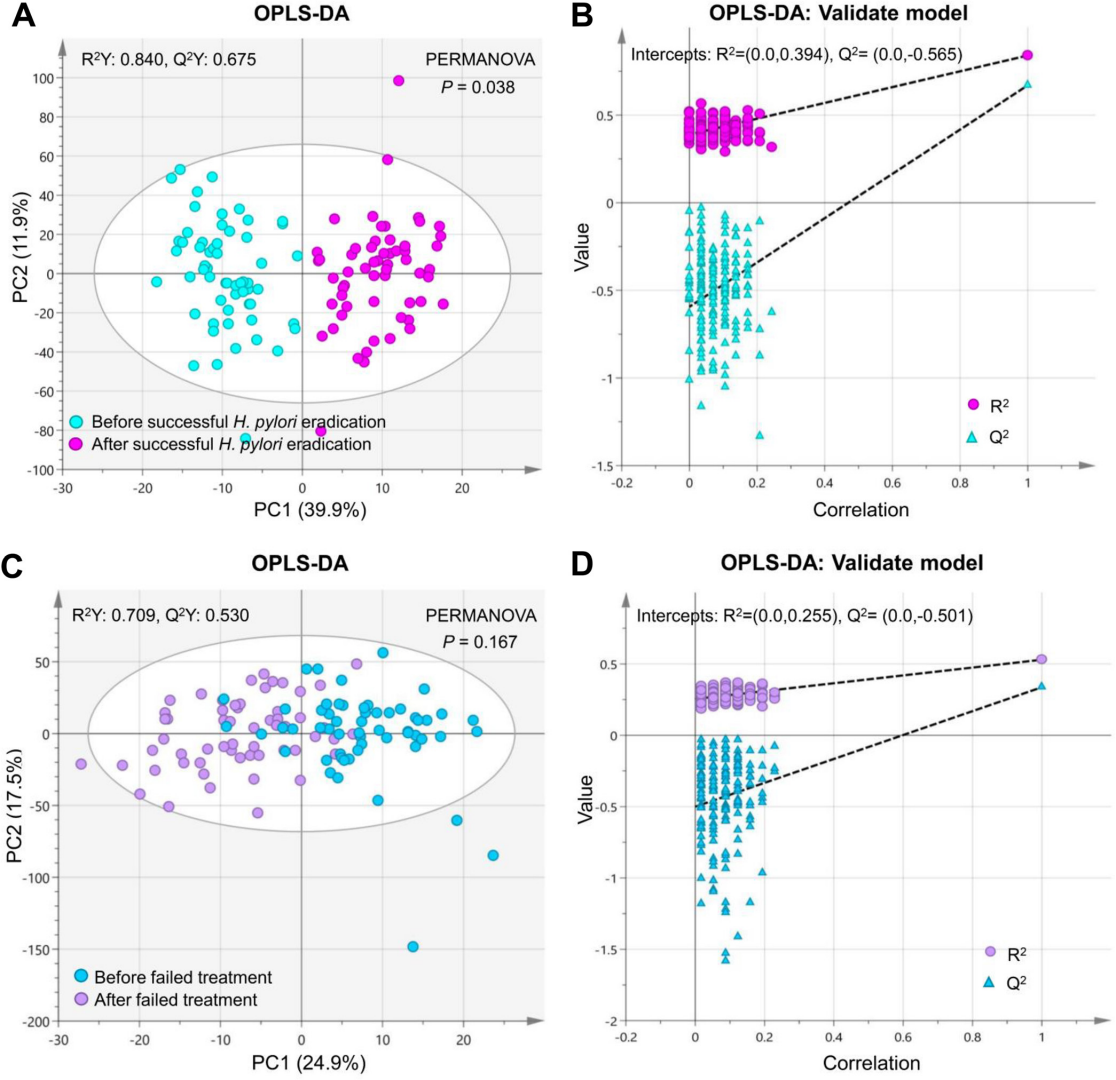
Metabolomics profiles in gastric biopsy specimens before and after anti-H. pylori treatment. Orthogonal partial least-squares discrimination analysis (OPLS-DA) found significant differences in gastric metabolomics profiles before and after successful H. pylori eradication (A); however, no significant changes were found for participants who failed to clear H. pylori by medical therapy (C). The OPLS-DA models in panels A and C were validated in panels B and D, respectively.

A total of 267 metabolites were quantified using the Biocrates P500 platform in 230 gastric biopsy specimens, including 58 pairs before and after successful eradication and 57 pairs before and after failed treatment. Orthogonal partial least-squares discrimination analysis (OPLS-DA) found significant differences in gastric metabolomics profiles before and after successful eradication (*R*^2^ = 0.84, *Q*^2^ = 0.68, and *P = *0.038 [Fig. 1A and B]). However, in the failed treatment group, gastric metabolomics profiles were not significantly changed by medical therapy (*R*^2^ = 0.71, *Q*^2^ = 0.53, and *P = *0.167 [Fig. 1C and D]).

A total of 81 metabolites showed significant alterations after successful eradication compared to failed treatment, with fold changes of >1.5 and *P* values of <0.05 adjusted for multiple comparison by the false-discovery rate (FDR) (Table S2). The differential metabolites include 4 acylcarnitines (Cx), 3 ceramides (Cer), 3 cholesterol esters (CE), 1 fatty acid (FA), 10 sphingolipids (SM), 6 triacylglycerols (TG), 44 glycerophospholipids, and 10 glycosylceramides. The glycerophospholipids and glycosylceramides can be further subdivided as 1 lyso-phosphatidylcholine (lysoPC), 43 phosphatidylcholines (PC), 5 hexosylceramides (HexCer), 3 dihexosylceramides (Hex2Cer), and 2 trihexosylceramides (Hex3Cer).

**FIG 2:**
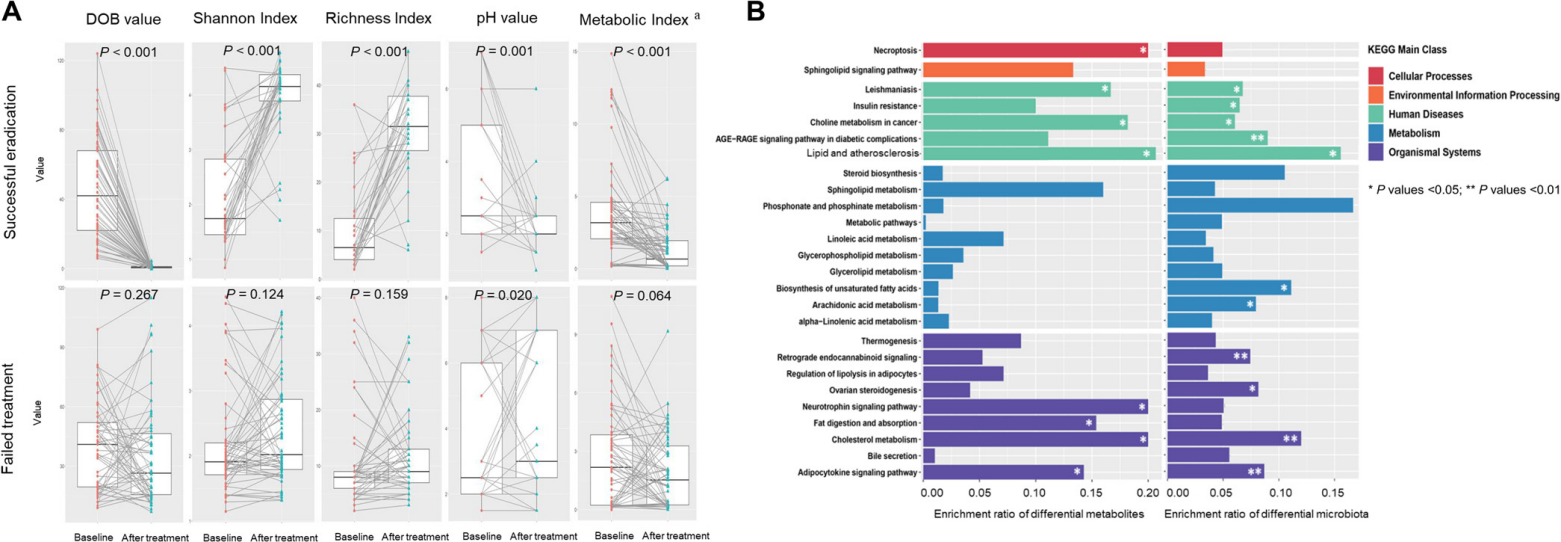
Potentially relevant influence factors and pathway enrichment analyses for gastric metabolite alterations after H. pylori eradication. (A) Paired plots showed significant decreasing trends of DOB values (*P < *0.001), pH values (*P = *0.001), and metabolic indexes (*P < *0.001) and significant increasing trends of microbial diversity indexes (*P < *0.001 for both) after successful H. pylori eradication. However, in the failed treatment group, except that pH values were increased significantly (*P = *0.020), the factors showed no significant alterations (*P > *0.05 for all). a, metabolic index was calculated using the 81 differential metabolites after H. pylori eradication to represent the overall metabolic status in each gastric biopsy specimen by the logistic regression equation in Materials and Methods. DOB, delta over baseline. (B) A total of 26 pathways were enriched by both the differential metabolites and microbiota after successful eradication. Among them, 5 pathways were significantly changed by eradication in both metabolomics and microbiome analyses, including pathways for leishmaniasis, adipocytokine signaling, lipid and atherosclerosis, choline metabolism in cancer, and cholesterol metabolism (*P* values <0.05 for all). *, *P* values < 0.05; **, *P* values < 0.01.

We investigated the potentially relevant influence factors for gastric metabolite alterations after H. pylori eradication. To represent the overall metabolic status in each gastric biopsy specimen, we calculated a comprehensive metabolic index using the 81 differential metabolites. Potentially relevant factors include DOB value in ^13^C-UBT representing H. pylori infection status, microbial Shannon and Richness indexes representing gastric microbial diversity, and gastric juice pH value. Figure 2A shows significant increases in microbial diversity indexes (*P* < 0.001 for both) and decreases in pH values (*P = *0.001) as well as in metabolic indexes (*P < *0.001) accompanying the dramatically decreasing trend of DOB values (*P < *0.001) after successful eradication. However, except for the fact that pH values were increased (*P = *0.020) in the failed treatment group, the factors showed no significant alterations (*P < *0.05 for all).

The putative functions of the 81 differential metabolites after H. pylori eradication were found to be enriched in 29 pathways using the Kyoto Encyclopedia of Genes and Genomes (KEGG) database. We compared the 29 metabolic pathways with the previously predicted microbiota functional-capacity changes (7) in the same intervention participants. A total of 26 pathways were enriched both by the differential metabolites and microbiota, covering 5 main classes, such as cellular processes, environmental information processing, human diseases, metabolism, and organismal systems (Fig. 2B). Among them, 5 pathways were significantly changed after eradication in both metabolomics and microbiome analyses, including leishmaniasis, adipocytokine signaling, lipid and atherosclerosis, choline metabolism in cancer, and cholesterol metabolism pathways (*P < *0.05 for all).

### Interactions between differential metabolites and microbiota before and after H. pylori eradication.

**FIG 3:**
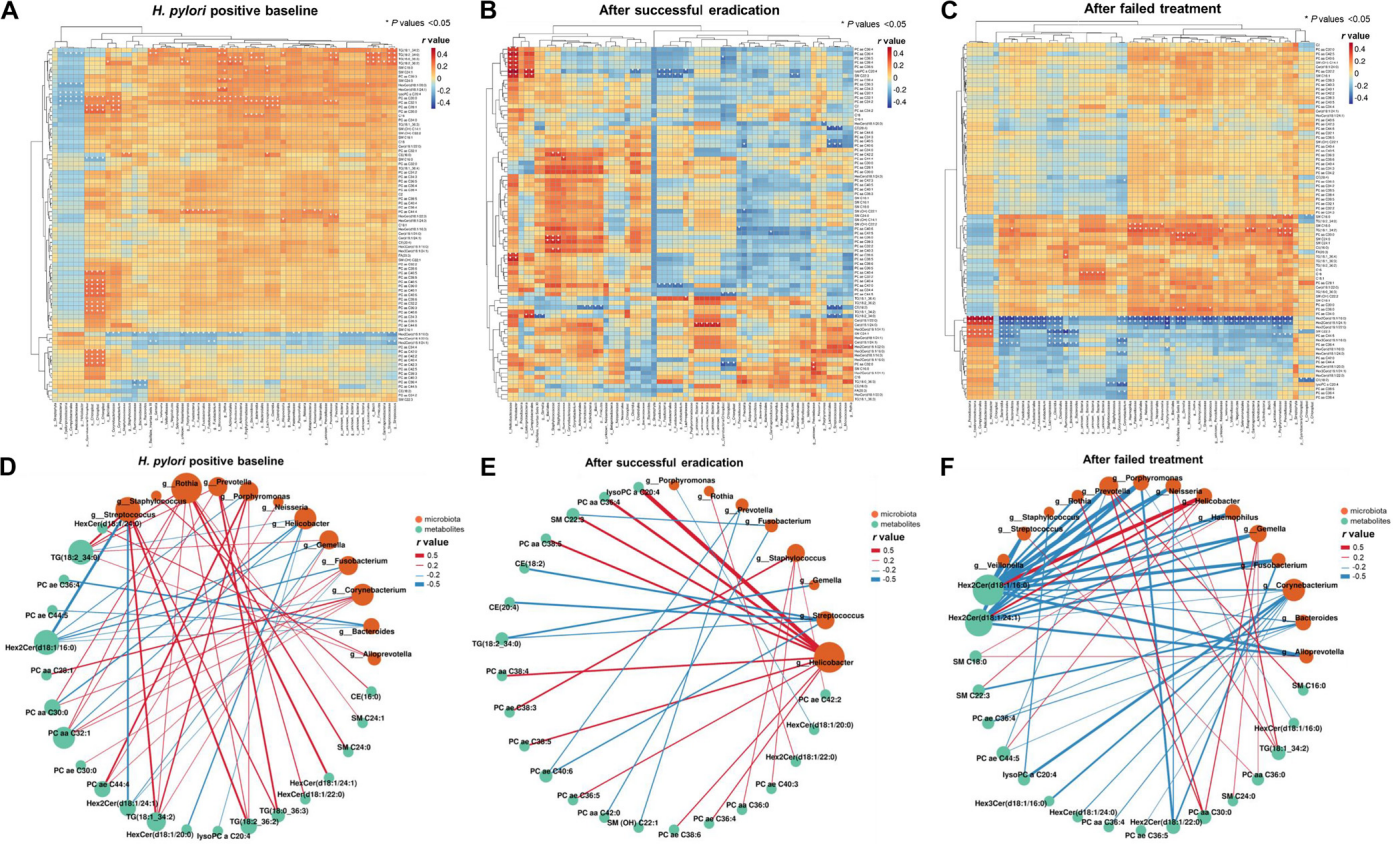
Interactions between differential metabolites and the microbiota before and after anti-H. pylori treatment. The heat maps show correlations between 81 differential metabolites and 65 previously identified differential bacterial taxa in biopsy specimens of subjects at H. pylori-positive baseline (A), after successful eradication (B), or after failed treatment (C). *, *P* values <0.05. The correlation networks showed the significant associations between differential metabolites and previously identified differential genera in biopsy specimens of subjects at H. pylori-positive baseline (D), after successful eradication (E), or after failed treatment (F). Red lines represent positive genus-metabolite correlations. Blue lines represent negative genus-metabolite correlations. Green nodes represent differential metabolites. Orange nodes represent gastric microbiota. Node radii are based on the number of significant genus-metabolite correlations. Cx, acylcarnitines; Cer, ceramides; CE, cholesterol esters; FA, fatty acids; HexCer, hexosylceramides; Hex2Cer, dihexosylceramides; Hex3Cer, trihexosylceramides; PC, phosphatidylcholines; lysoPC, lyso-phosphatidylcholine; SM, sphingomyelins; TG, triacylglycerols.

In our microbiome analysis, 65 bacterial taxa were significantly changed after successful H. pylori eradication (7). We used Spearman’s correlation analysis to assess the potential interplays between the 81 differential metabolites and the 65 differential taxa in the 91 participants with both microbiome and metabolomics results. We found 71 negative (−0.29 < *r <* −0.21; *P < *0.05 for all) and 177 positive (0.21< *r < *0.28; *P < *0.05 for all) significant correlations between 34 metabolites and 58 taxa in H. pylori-positive baseline biopsy specimens (Fig. 3A). After successful eradication, stronger correlations were found between 30 metabolites and 41 taxa, including 49 negative (−0.44 < *r <* −0.34; *P < *0.05 for all) and 44 positive (0.34 < *r < *0.52; *P < *0.05 for all) correlations (Fig. 3B). After failed treatment (Fig. 3C), 20 metabolites and 63 taxa were significantly correlated, with 163 negative associations (−0.52 < *r <*−0.26; *P < *0.05 for all) and 60 positive associations (0.26 < *r < *0.48; *P < *0.05 for all).

Of the 65 differential taxa, we further focused on the 18 differential genera after H. pylori eradication (7). Significant interactions between differential genera and metabolites were visualized by correlation network construction. *Helicobacter* was negatively correlated with glycerophospholipids, glycosylceramide, and triacylglycerol (−0.24 < *r <* −0.21; *P < *0.05 for all) in baseline biopsy specimens (Fig. 3D), while it was positively correlated with glycerophospholipids and sphingolipids (0.37 < *r < *0.51; *P < *0.05 for all) after successful eradication (Fig. 3E). Positive correlations between triacylglycerols and *Prevotella* (0.21<*r < *0.26; *P < *0.05 for all) and negative correlations between glycosylceramides and many non-*Helicobacter* genera, such as *Fusobacterium*, *Gemella*, and Streptococcus (−0.29 < *r <* −0.20; *P < *0.05 for all), were notable in baseline biopsy specimens (Fig. 3D) but absent after successful eradication (Fig. 3E). After failed treatment (Fig. 3F), more negative correlations were identified between differential genera and metabolites (−0.52 < *r <* −0.26; *P < *0.05 for all).

### Interactions between differential genera and metabolites in gastric lesions.

**FIG 4:**
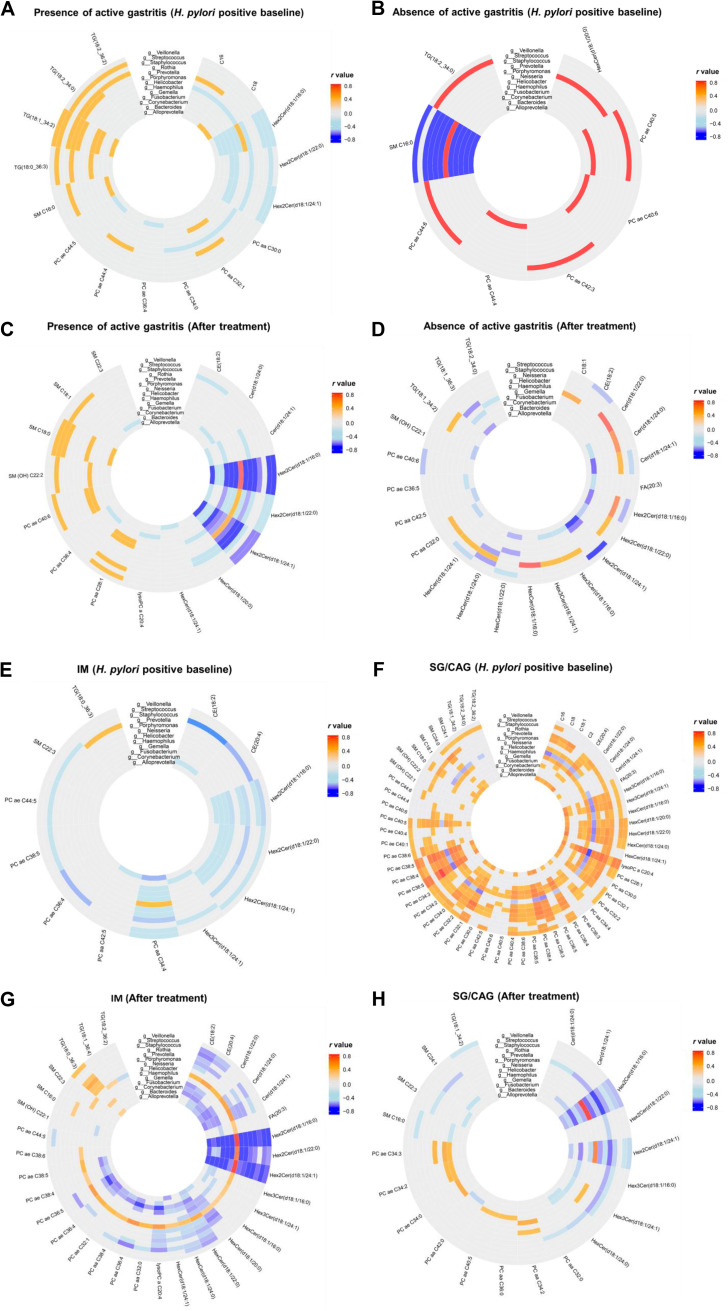
Interactions between differential metabolites and genera in various gastric lesions. The significant correlations between differential metabolites and genera are shown for H. pylori-positive baseline biopsy specimens with presence (A) or absence (B) of active gastritis and in biopsy specimens after treatment in the presence (C) or absence (D) of active gastritis. The significant correlations between differential metabolites and genera are also shown for H. pylori-positive baseline biopsy specimens with IM (E) or SG/CAG lesions (F) and biopsy specimens after treatment with IM (G) or SG/CAG lesions (H).

Besides anti-H. pylori treatment, some specific genus-metabolite interactions are also associated with gastric lesions. In baseline biopsy specimens, significant negative correlations of glycosylceramides (−0.30 < *r* < −0.23; *P < *0.05 for all) with some non-*Helicobacter* genera, such as *Fusobacterium*, *Gemella*, *Porphyromonas*, and Streptococcus (Fig. 4A), were found when active gastritis was present, while they were not found when active gastritis was absent (Fig. 4B). After anti-H. pylori treatment, we still observed more negative correlations of glycosylceramides with these non-*Helicobacter* genera (−0.61 < *r <* −0.26; *P < *0.05 for all) in active gastritis biopsy specimens (Fig. 4C) than in biopsy specimens without active gastritis (Fig. 4D). Positive correlations of glycosylceramides were only found with *Helicobacter* (0.31 < *r *< 0.58; *P < *0.05 for all) after treatment.

We further identified some characteristic genus-metabolite correlations in IM compared to mild lesions (superficial gastritis [SG]/chronic atrophic gastritis [CAG]). In baseline biopsy specimens, many negative correlations were found between differential metabolites, such as glycosylceramides, and *Fusobacterium*, *Gemella*, *Neisseria*, and Streptococcus (−0.49 < *r* < −0.28; *P < *0.05 for all) in IM lesions (Fig. 4E). In contrast, no significant negative correlation was found between metabolites and the non-*Helicobacter* genera in mild baseline lesions (Fig. 4F). Similarly, more significant negative correlations (−0.63 < *r* < −0.25; *P < *0.05 for all) were found in IM lesions after intervention between glycerophospholipids, glycosylceramides, and ceramides and various non-*Helicobacter* genera (Fig. 4G) than in SG/CAG lesions (Fig. 4H). Positive correlations (0.25 < *r *< 0.71; *P < *0.05 for all) between *Prevotella* and triacylglycerol and between *Helicobacter* and various differential metabolites were also noticed in IM lesions.

### Screening of significant gastric metabolites and their associated genera for gastric lesion progression.

**FIG 5:**
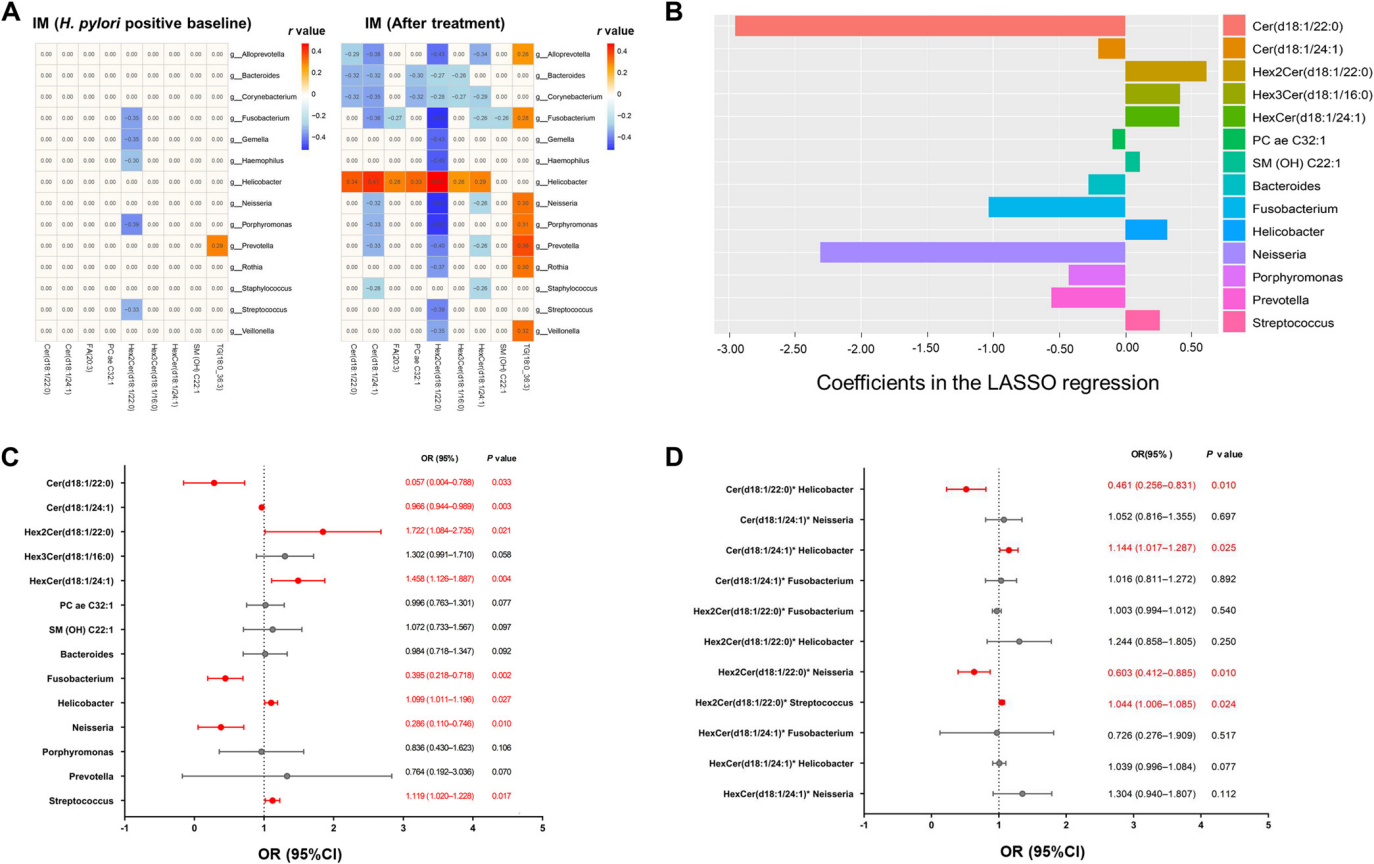
Screening of significant gastric metabolites and their associated genera for gastric lesion progression. (A) The 9 differential metabolites in IM lesions before or after interventions showed 49 pairs of significant correlations with 14 differential gastric genera. (B) Alterations of 9 metabolites and 14 associated genera were screened by LASSO regression, with 7 metabolites and 7 genera showing significant associations with risk of lesion progression to IM. (C) These significantly altered metabolites and genera were selected by multivariate unconditional logistic regression adjusted for age, gender, and the effect of anti-H. pylori treatment with Cer(d18:1/22:0), Cer(d18:1/24:1), Hex2Cer(d18:1/22:0), HexCer(d18:1/24:1) and *Fusobacterium*, *Helicobacter*, *Neisseria*, and Streptococcus showing significant associations with risk of lesion progression to IM. (D) The selected 4 metabolites, 4 genera, and their 11 significant interactions according to panel A were further enrolled in a multivariate unconditional logistic regression model with 4 genus-metabolite interactions showing significant associations with risk of lesion progression to IM.

To identify significant metabolites and their associated genera in advanced gastric lesions, we preliminarily selected 13 and 33 differential metabolites correlated with the differential genera in baseline and follow-up IM biopsy specimens after intervention, respectively (Tables S3 and S4). A total of 35 genus-associated metabolites (11 overlapping between baseline and follow-up biopsy specimens) were compared between the IM and SG/CAG groups by multivariate regression. In baseline biopsy specimens (Table S5), 6 metabolites showed significant differences between the IM and SG/CAG groups, including Cer(d18:1/24:1), FA(20:3), PCaeC32:1, HexCer(d18:1/24:1), SM(OH)C22:1, TG(18:0_36:3), all *P < *0.05. In follow-up biopsy specimens (Table S6), Cer(d18:1/22:0), Hex2Cer(d18:1/22:0), and Hex3Cer(d18:1/16:0) further showed significant differences between IM and mild lesions (*P < *0.05 for all). The 9 differential metabolites were remarkably associated with 14 differential genera, showing 49 pairs of genus-metabolite correlations in IM subjects before and after interventions (Fig. 5A).

In the 6-month follow-up period after eradication, we identified 21 high-risk subjects who progressed from SG/CAG to IM and 23 low-risk subjects who reversed from IM to mild lesions (8 subjects) or remained as showing SG/CAG (15 subjects). We further evaluated the alterations of the 9 metabolites and 14 associated genera with the risk of lesion progression by least absolute shrinkage and selection operator (LASSO) regression. A total of 7 metabolites and 7 genera were significantly associated with the risk of progression to IM, including Cer(d18:1/22:0), Cer(d18:1/24:1), Hex2Cer(d18:1/22:0), Hex3Cer(d18:1/16:0), HexCer(d18:1/24:1), PCae32:1, and SM(OH)C22:1 and *Bacteroides*, *Fusobacterium*, *Helicobacter*, *Neisseria*, *Porphyromonas*, *Prevotella*, and Streptococcus (Fig. 5B). These significantly altered metabolites and genera were selected by multivariate unconditional logistic regression adjusted for age, gender, and effect of intervention. Finally, 4 metabolites and 4 genera showed significant associations with the risk of progression to IM, including Cer(d18:1/22:0), Cer(d18:1/24:1), Hex2Cer(d18:1/22:0), and HexCer(d18:1/24:1) and *Fusobacterium*, *Helicobacter*, *Neisseria*, and Streptococcus (Fig. 5C).

The 4 metabolites, the 4 genera, and their 11 significant genus-metabolite interactions according to Fig. 5A were further enrolled in a multivariate logistic regression model to identify significant interactions by comparing high-risk and low-risk subjects for progression to IM. Four significant genus-metabolite interactions were identified, including Cer(d18:1/22:0)-*Helicobacter*, Cer(d18:1/24:1)-*Helicobacter*, Hex2Cer(d18:1/22:0)-Streptococcus, and Hex2Cer(d18:1/22:0)-*Neisseria* (Fig. 5D).

### Improved discrimination of gastric lesion progression by genus-metabolite interactions.

**FIG 6:**
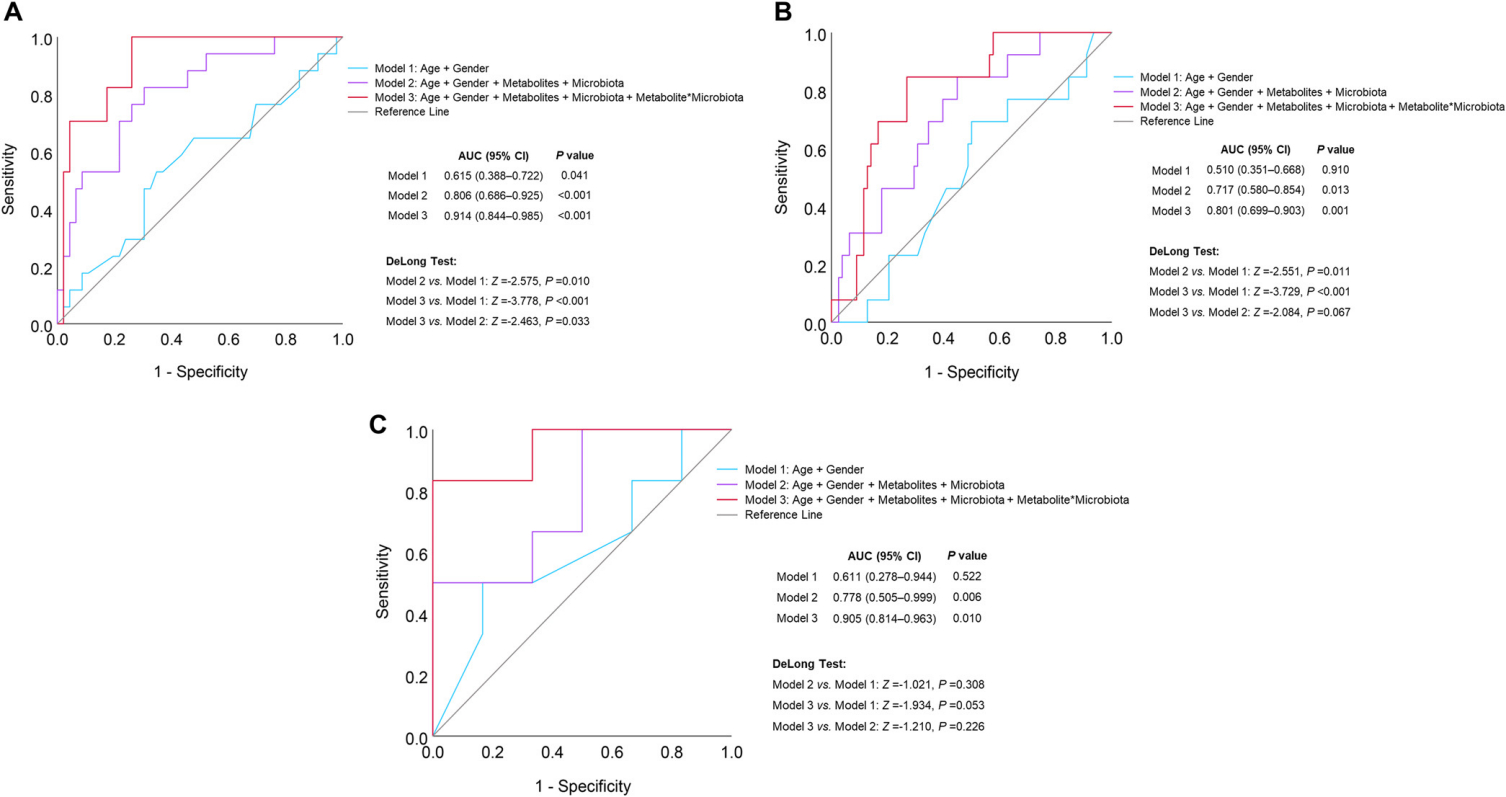
Improved discrimination of gastric lesion progression by genus-metabolite interactions. ROC curve analysis was conducted to compare the discrimination performances of three different models between 21 high-risk subjects who progressed from mild gastric lesions (SG/CAG) to IM and 23 low-risk subjects who reversed from IM to mild lesions or remained with mild lesions in short-term follow-up period (A) or between 13 high-risk subjects who progressed to or remained with IM/LGIN and 6 low-risk subjects who remained with SG/CAG in the long-term follow-up period (B). In particular, the performances of the 3 models were also compared between 6 high-risk subjects who progressed to LGIN and 6 low-risk subjects who remained as showing SG/CAG in the long-term follow-up period (C). The discrimination performances of the 3 models were verified by random forest with leave-one-out cross-validation and compared by the DeLong test.

Receiver operating characteristic (ROC) curve analysis between high-risk and low-risk subjects regarding lesion progression was conducted. The discrimination performances were compared among model 1 (including age and gender), model 2 (including age, gender, 4 metabolites, and 4 genera) and model 3 (including age, gender, 4 metabolites, 4 genera, and 4 significant genus-metabolite interactions). The area under the curve (AUC) was remarkably increased, from 0.651 (*P = *0.041) by model 1 to 0.806 (*P < *0.001) by model 2. Furthermore, model 3 can significantly improve the performance of model 2 by adding 4 genus-metabolite interactions, with an AUC of 0.914 (*P < *0.001). The discrimination performances of the 3 models were verified by random forest with leave-one-out cross-validation and compared by the DeLong test, with significant differences between model 2 and model 1 (*P = *0.010), as well as between model 3 and model 2 (*P = *0.033) (Fig. 6A).

In the high-risk and low-risk subjects for lesion progression to IM after the 6-month follow-up, 19 subjects were further followed once by endoscopic screening from 2018 to 2021. Among them, 13 high-risk subjects progressed to or remained as showing IM/low-grade intraepithelial neoplasia (LGIN) and 6 low-risk subjects remained as SG/CAG in the long-term follow-up period. Model 2 with short-term metabolite and genus alterations and model 3 with additional genus-metabolite interactions still can discriminate the high-risk and low-risk subjects for lesion progression in long-term follow-up, with AUCs of 0.717 (*P = *0.013) and 0.801 (*P = *0.001), respectively (Fig. 6B). In particular, the 6 high-risk subjects who progressed to LGIN in long-term follow-up (Fig. 6C) were well distinguished from the 6 low-risk subjects by model 2 (AUC = 0.778 and *P = *0.006) and model 3 (AUC = 0.905 and *P = *0.010).

## DISCUSSION

Our previous prospective studies revealed that H. pylori infection can induce gastric microbial dysbiosis, which may be involved in carcinogenesis (7, 8). The present study further profiled the microbiota-associated metabolite alterations in gastric mucosa in the same group of intervention participants. We found that H. pylori eradication can significantly change gastric metabolites and their interactions with the microbiota. The interactions between the gastric microbiota and metabolites may play a role in the progression of precancerous lesions.

Numerous studies have deeply investigated the molecular characteristics of GC tissues by multiomics technologies (13–15). However, it is still very difficult to reveal the dynamic mechanisms in the natural evolution process of precancerous lesions. Our previous anti-H. pylori intervention and follow-up study provides a good opportunity to assess the H. pylori-associated metabolite alterations in early stage of precancerous development. By comparing the metabolic profiles before and after treatment, we found significant changes in successful eradication group rather than in failed treatment group. Furthermore, 81 metabolites were significantly altered by successful eradication. Some significant metabolites in our study, such as fatty acid and glycerophospholipids, are consistent with those in a GC-associated metabolomics study (12), which suggests that H. pylori-associated metabolite alterations in the gastric mucosa may be involved in progression of lesions and carcinogenesis.

Integrative studies of metagenomics and metabolomics have highlighted the importance of the microbiota and metabolite interactions in gastrointestinal carcinogenesis (11, 16, 17). Our study also investigated possible relevant factors for metabolite alterations by H. pylori eradication. Dramatic alterations in gastric microbial diversity and metabolic indices were found simultaneously after successful eradication. However, such significant changes were not seen after failed treatment. These phenomena further imply that the metabolite alterations after H. pylori eradication may be associated with the comprehensive microbial changes in the gastric mucosa.

KEGG enrichment analysis can help to better understand the possible functions of the differential metabolites by H. pylori eradication. A total of 29 metabolic pathways were enriched by the 81 differential metabolites, including 26 consistent pathways with the predicted functional-capacity changes by the differential microbiota in the same intervention participants (7). The 5 pathways significantly changed by H. pylori eradication in both metabolomics and microbiome analyses include pathways for leishmaniasis, adipocytokine signaling, lipid and atherosclerosis, choline metabolism in cancer, and cholesterol metabolism. Our results support the importance of cholesterol-rich lipid rafts on the cell membrane for bacterial pathogen or virulence factor adhesion in the H. pylori infection process (18). On the other hand, the significant alterations of lipid and cholesterol metabolism pathways after H. pylori eradication may also be associated with lipid metabolism reprogramming and lipoprotein-mediated cholesterol entry in GC progression (19). However, further validation of such mechanisms is needed.

H. pylori infection and eradication were reported to be associated with gut microbiome-metabolome interactions and alterations of serum metabolites (20–22). However, the effects of H. pylori infection on the interplays between the gastric microbiota and metabolites still remain unclear. In the present study, we found many significant correlations between the differential microbiota and metabolites in H. pylori-positive baseline biopsy specimens, which can be changed by eradication. The dominating positive correlations in baseline were converted to relatively more negative correlations after treatment. *Helicobacter* was notable for negative correlations with glycerophospholipids, glycosylceramides, and triacylglycerols in baseline but for positive correlations with glycerophospholipids and sphingolipids after treatment.

In addition to anti-H. pylori treatment, gastric lesions are also associated with microbiota-metabolite interactions. The characteristic negative correlations between glycosylceramides and *Fusobacterium*, Streptococcus, and *Gemella* in H. pylori-positive baseline biopsy specimens were also noticed in active gastritis and IM lesions compared to respective references. Furthermore, positive correlations between *Prevotella* and triacylglycerols or between *Helicobacter* and many differential metabolites, including ceramides, cholesterol esters, fatty acid, and glycerophospholipids, were found specifically in IM compared to SG/CAG subjects. A previous study also found similar positive correlations between *Helicobacter* and fatty acids or phosphatidylcholines in GC tissues (12), which suggested potential roles of microbiota-metabolite interactions in gastric carcinogenesis. However, distinctive positive correlations of *Lactobacillus* and Streptococcus with the metabolites in the pathway of glutathione, cysteine, and methionine metabolism were identified in GC tissues (12), which were not found in precancerous lesions in our study. The combination of the above-described findings suggests that some specific gastric microbiota-metabolite interactions may be involved in gastric inflammation and carcinogenesis, while longitudinal observations are still needed to deeply understand the temporal and causal relationships.

An integrative study has combined gut bacteria and metabolite markers to improve the diagnostic performance among healthy, colorectal adenoma, and cancer subjects, indicating microbiota and metabolite interactive mechanisms along the adenoma-carcinoma sequence (11). Our prospective validation further confirmed that some significant metabolites and their associated genera in IM lesions from baseline or follow-up points were altered remarkably when the mild lesions progressed to IM. Especially, the panel containing Cer(d18:1/22:0), Cer(d18:1/24:1), Hex2Cer(d18:1/22:0), and HexCer(d18:1/24:1), *Fusobacterium*, *Helicobacter*, *Neisseria*, and Streptococcus, and 4 significant genus-metabolite interactions can distinguish high-risk subjects who progressed to IM/LGIN in short-term or even in long-term follow-up periods.

Ceramides and glycosylceramides, the metabolites associated with progression of gastric lesions in the present study, were found to be important for their involvement in various signaling pathways related to cell survival and senescence (23, 24). Ceramides are interconvertible to glycosylceramides under the mediation of glucosylceramide synthase, which may play roles in the pathology of cancer, diabetes, and infectious diseases (25–27). Many bacteria, including *Neisseria*, *Bacteroides*, *Porphyromonas*, and *Prevotella*, are associated with synthesis and conversion of ceramides and their derivatives (28–30). Our findings together with this evidence support the notion that interactions between the microbiota and metabolites may promote gastric lesion progression, although further validations are still needed.

Our study has several strengths. In contrast to previous case-control studies, our prospective design with self-comparisons before and after successful and failed treatment or gastric lesion progression can effectively control the complex confounding factors and investigate the effects of H. pylori infection or lesion progression on metabolites and their interactions with the microbiota. The simultaneous collection of the two fresh biopsy specimens from adjacent stomach sites for metabolomics and microbiome detections can guarantee the feasibility and rationality of the integrative analysis. However, limitations of our study include a modest sample size before and after intervention and a relatively short follow-up period with no lesions having progressed to GC. In the present study, we targeted and quantified 630 metabolites in gastric biopsy specimens using the Biocrates MxP Quant 500 kit; however, a broader spectrum of metabolic profiling is needed in the future. Furthermore, functional investigations in human cell lines or animal models are needed for a deeper understanding of the biological mechanisms for the complicated metabolic regulations among H. pylori, other gastric bacteria, and host epithelial cells.

In conclusion, our study found that successful H. pylori eradication can significantly alter gastric metabolites and their interactions with the microbiota. Some microbiota-metabolite correlations can be found specifically in active gastritis and IM lesions, such as negative correlations between glycosylceramides and *Fusobacterium*, *Gemella*, and Streptococcus. A panel including differential metabolites, genera, and their interactions may be associated with the risk of gastric lesion progression to IM/LGIN. Our findings provide new insights into the metabolite and microbiota interactions in H. pylori-associated gastric lesion progression.

## MATERIALS AND METHODS

### Patient and public involvement.

The present study was conducted within the framework of the National Upper Gastrointestinal Cancer Early Detection Project in Linqu County, Shandong Province, China. This county possesses one of the highest GC mortality rates worldwide (age adjusted rates per 100,000 were 55 for men and 19 for women from 1980 to 1982) (31).

In December 2016, 332 project volunteers were screened using ^13^C-UBT, with 186 subjects reported as H. pylori positive and 146 as negative. The positive subjects were invited for a 10-day quadruple anti-H. pylori treatment including omeprazole (20 mg twice daily), tetracycline (750 mg three times daily), metronidazole (400 mg three times daily), and bismuth citrate (300 mg twice daily). Six months after the treatment, participants were followed up by repeated ^13^C-UBT testing, endoscopic examination, and drug intake/adverse effect interview. The participants with completed drug intake records and positive follow-up results of ^13^C-UBT testing were defined as failed treatment. A total of 145 participants completed baseline and follow-up endoscopic examinations and agreed to provide gastric biopsy specimens and general health information using a structured questionnaire on age, sex, cigarette and alcohol consumption habits, and antibiotic use history (use of any kind of antibiotic at least 1 day within 6 months before the baseline interview).

For the present study, 115 anti-H. pylori treatment participants were enrolled (58 subjects with successful and 57 subjects with failed treatment) for possessing eligible paired baseline and follow-up fresh gastric biopsy specimens. For each subject, at least two fresh biopsy specimens were collected from the lesser curve of antrum and angular incisure, one for the previously completed 16S rRNA gene sequencing (7) and the other one for the targeted quantitative metabolomics detection. This study was approved by the institutional review boards of Peking University Cancer Hospital and Institute (2020KT145), and written informed consent was obtained from all of the participants.

### Upper endoscopic examination and histopathology.

Upper endoscopic examinations were conducted by two experienced gastroenterologists using video endoscopes (Olympus). Gastric mucosa was examined and one biopsy specimen was obtained from the lesser curve of the antrum for pathological diagnosis according to the same standard operating procedure in baseline as well as follow-up examinations. The gastric mucosa specimens were reviewed blindly by two pathologists according to the criteria proposed by the Chinese Association of Gastric Cancer (32) as normal or showing SG, CAG, IM, low-grade intraepithelial neoplasia (LGIN), or high-grade intraepithelial neoplasia (HGIN) based on the most severe histology. Gastritis status was diagnosed according to the updated Sydney System (33) as presence or absence of active gastritis.

### Metabolomics profiling.

We identified differential metabolites after H. pylori eradication utilizing the Biocrates MxP Quant 500 kit (Biocrates, Innsbruck, Austria), which is capable of quantifying 630 metabolites across the metabolic spectrum and based on a quality-controlled, highly reproducible analysis platform. The frozen gastric biopsy specimens were weighed and homogenized according to the guidelines of the manufacturer. The homogenates were centrifuged at 2 to 4°C. Supernatants were transferred to a 96-well-based Biocrates sample preparation plate which was impregnated with internal standards and dried under a nitrogen stream. Then 5% phenylisothiocyanate (PITC) solution was added for derivatization. After the derivatization, the target analytes were extracted with an organic solvent. The obtained extracts were analyzed for lipids by flow injection analysis tandem mass spectrometry (FIA-MS/MS) and small molecules by liquid chromatography-tandem mass spectrometry (LC-MS/MS).

### Metabolomics data analysis.

The raw data were normalized and assessed for robustness, and the concentrations of all the metabolites were calculated using internal standards and quality control samples by MetLIMS software (https://nml.unbs.go.ug), R statistical software (4.0.2; R Foundation for Statistical Computing), and online versions of MetaboAnalyst (http://www.metaboanalyst.ca) (34). Orthogonal partial least-squares discrimination analysis (OPLS-DA) was performed using SIMCA-P software v.14.1 (Umetrics, Umea, Sweden). *P* values in the OPLS-DA plot were calculated by permutational multivariate analysis of variance (PERMANOVA) for dissimilarities between biopsy specimens before and after treatment. The differential metabolites after H. pylori eradication were selected according to *P* values adjusted by false-discovery rate (FDR) and fold changes as the ratios of the average metabolite alterations by successful eradication to the average metabolite alterations by failed treatment. Metabolites with *P* values of <0.05 and fold changes of >1.5 were considered significant. To represent the overall metabolic status, we calculated a comprehensive metabolic index using the 81 differential metabolites after H. pylori eradication in each gastric biopsy specimen by the following logistic regression equation:

$$\text{Metabolic index}=\beta_{0}\;+{\beta \;}_{1}x_{1}\;+\;\beta_{2}x_{2}+\;\ldots \;+\beta_{80}x_{80}\;+\;\beta_{81}x_{81}$$

### 16S rRNA gene sequencing and data analysis.

DNA was extracted from biopsy specimens using the QIAamp DNA minikit (Qiagen, CA, USA). Details of amplification, sequencing, raw data processing, and differential taxon selection were described previously (7). Briefly, the V3-V4 region of microbial 16S rRNA gene was amplified using universal primers (341F, 5′-CCTACGGGNBGCASCAG-3′; 805R, 5′-GACTACNVGGGTATCTAATCC-3′) and sequenced on the Illumina HiSeq 2500 PE250 platform. Sequence reads were processed and clustered into operational taxonomic units (OTUs) using IMNGS (www.imngs.org). Differential microbial taxa with relative abundances of >1% after eradication were selected by paired *t* tests when *P* values adjusted by FDR were less than 0.05.

### Prediction functions of the differential metabolites and microbiota.

The differential metabolites and microbiota after H. pylori eradication identified in our present study and previous study (7) were subjected to metabolic pathway enrichment analysis using the Kyoto Encyclopedia of Genes and Genomes (KEGG) database and three online analytical platforms (http://www.metaboanalyst.ca, https://www.microbiomeanalyst.ca, and http://kobas.cbi.pku.edu.cn/) (11, 12, 17, 35, 36).

### Statistical analyses.

The Mann-Whitney U test was used to compare the metabolite alterations between successful and failed treatment groups. The significant biomarkers in IM or high-risk subjects for lesion progression were identified by logistic regression analysis adjusted by age, gender, or effect of treatment. Microbiota-metabolite correlations were evaluated using the rank correlation analysis of the Spearman algorithm. To build a panel of biomarkers for discrimination of high-risk gastric lesion progression subjects, differential metabolites and genera were selected by the least absolute shrinkage and selection operator (LASSO) regression. Receiver operating characteristic (ROC) curve analysis was used to assess the discrimination performance of significant metabolites, genera, and their interactions for high-risk progression subjects, which was verified by random forest model with leave-one-out cross-validation and compared by the DeLong test. Statistical significance was considered when *P* values or adjusted *P* values were below 0.05 (two tailed).

### Data availability.

The high-throughput sequencing and large data sets of microbiome and metabolomics for the analysis in the present study can be obtained from the corresponding author for reuse under the permission of the Chinese Human Genetic Resources Administration Office.
