# Supplementary material for: Metabolite Alterations and Interactions with Microbiota in Helicobacter pylori-Associated Gastric Lesions
Source: Microbiol Spectr. 2023 Jun 26;11(4):e05347-22. doi: 10.1128/spectrum.05347-22 (PMC10434277; doi:10.1128/spectrum.05347-22)
Supplement: Supplemental file 1 — Supplemental material. Download spectrum.05347-22-s0001.pdf, PDF file, 0.5 MB [file spectrum.05347-22-s0001.pdf]

Metabolite alterations and interactions with microbiota in Helicobacter pylori associated gastric lesions

Lei Peng,[a] Yang Guo,[a] Markus Gerhard,[b,c,d] Juan-Juan Gao,[a] Zong-Chao Liu,[a] Raquel Mejías-Luque,[b,c,d] Lian Zhang,[a] Michael Vieth,[b,e] Jun-Ling Ma,[a] Wei-Dong Liu,[f] Zhe-Xuan Li,[a,b] Tong Zhou,[a] Wen-Qing Li,[a,b] Wei-Cheng You,[a,b] Yang Zhang,[a,b] Kai-Feng Pan[a,b]

Supplementary Table S1. Baseline characteristics of anti-*H. pylori* intervention participants

Baseline characteristics	Successful eradication group (n = 58)	Failed treatment group (n = 57)	P value
Age, years (Mean ± SD) [a]	56.6 ± 9.2	56.2 ± 8.9	0.801
DOB value in ^{13}C-UBT (Median, P_{25}–P_{75}) [b]	42.0 (21.5–68.3)	41.0 (19.0–52.0)	0.318
Gender (%) [c]			0.043
Male	30 (51.7%)	40 (70.2%)	
Female	28 (48.3%)	17 (29.8%)	
BMI, kg/m^2 (Mean ± SD) [a]	26.3 ± 1.2	26.2 ± 1.3	0.739
Smoking habits (%) [c]			0.720
No	41 (70.7%)	42 (73.7%)	
Yes	17 (29.3%)	15 (26.3%)	
Alcohol consumption (%) [c]			0.066
No	43 (74.1%)	33 (57.9%)	
Yes	15 (25.9%)	24 (42.1%)	
Gastric inflammation status (%) [c]			0.090
Absence of active gastritis	6 (10.3%)	6 (10.5%)	
Presence of active gastritis	52 (89.7%)	46 (80.7%)	
Missing	0 (0.0%)	5 (8.8%)	
Gastric lesion status (%) [d]			0.090
SG/CAG	30 (51.7%)	23 (40.4%)	
IM	28 (48.3%)	30 (52.6%)	
Missing	0 (0.0%)	4 (7.0%)	

[a] *t*-test

[b] Mann-Whitney U test

[c] χ^2 test

[d] Fisher's Exact Test

BMI, body mass index; CAG, chronic atrophic gastritis; ^{13}C-UBT, ^{13}C-urea breath test; DOB, Delta over baseline-value; *H. pylori*, *Helicobacter pylori*; IM, intestinal metaplasia; SG, superficial gastritis

Supplementary Table S2. Significantly altered metabolites after successful *H. pylori* eradication compared to failed treatment group

Metabolite	Metabolite change after failed treatment [b]	Metabolite change after successful eradication [a]	|FC| [c]	*P* value [d]
C16	0.017	-0.057	3.353	0.007
C2	0.029	-0.188	6.483	0.009
C18:1	0.008	-0.028	3.500	0.019
C18	0.006	-0.022	3.667	0.045
Cer(d18:1/24:0)	-0.066	-0.461	6.985	0.002
Cer(d18:1/22:0)	-0.023	-0.291	12.652	0.002
Cer(d18:1/24:1)	-0.151	-0.852	5.642	0.008
CE(18:2)	2.376	-9.561	4.024	0.005
CE(20:4)	-0.295	-4.270	14.475	0.008
CE(16:0)	0.192	-1.006	5.240	0.041
FA(20:3)	8.017	-29.961	3.737	<0.001
PC ae C38:6	0.323	-0.624	1.932	<0.001
PC ae C38:5	0.553	-1.250	2.260	<0.001
PC ae C36:5	0.492	-1.156	2.350	<0.001
PC ae C38:4	0.338	-0.845	2.500	<0.001
PC aa C36:4	3.374	-10.015	2.968	<0.001
PC aa C38:4	2.521	-5.392	2.139	<0.001
PC aa C40:5	0.126	-0.256	2.032	<0.001
PC aa C32:1	0.625	-1.003	1.605	<0.001
PC aa C36:5	0.084	-0.196	2.333	<0.001
PC aa C38:5	0.462	-1.615	3.496	<0.001
PC aa C38:6	0.371	-0.904	2.437	<0.001
PC aa C40:4	0.074	-0.198	2.676	<0.001
PC aa C38:3	1.134	-2.301	2.029	<0.001
PC ae C40:4	0.068	-0.186	2.735	<0.001
PC aa C32:0	5.702	-12.414	2.177	<0.001
PC ae C30:0	0.175	-0.291	1.663	<0.001
PC aa C34:3	0.167	-0.387	2.317	<0.001
PC ae C32:2	0.058	-0.163	2.810	<0.001
PC ae C32:1	0.334	-1.745	5.225	<0.001
PC ae C36:4	0.384	-0.995	2.591	<0.001
PC aa C32:2	0.055	-0.179	3.255	<0.001
PC aa C34:4	0.042	-0.144	3.429	<0.001
PC ae C40:5	0.053	-0.149	2.811	<0.001

PC aa C36:3	1.642	-3.353	2.042	<0.001
PC ae C34:3	0.215	-0.411	1.912	<0.001
PC ae C34:2	0.609	-0.967	1.588	<0.001
lysoPC a C20:4	0.390	-1.184	3.036	<0.001
PC ae C40:1	0.067	-0.106	1.582	<0.001
PC ae C34:0	0.438	-0.729	1.664	<0.001
PC aa C30:0	0.511	-1.078	2.110	<0.001
PC aa C36:0	0.068	-0.177	2.603	<0.001
PC ae C44:6	0.018	-0.121	6.722	<0.001
PC aa C34:2	6.509	-10.735	1.649	<0.001
PC ae C38:3	0.040	-0.215	5.375	0.001
PC ae C42:3	0.022	-0.130	5.909	0.001
PC ae C44:5	0.048	-0.167	3.479	0.001
PC aa C40:6	0.072	-0.186	2.583	0.001
PC ae C42:2	0.020	-0.061	3.050	0.003
PC aa C42:5	0.028	-0.049	1.750	0.004
PC ae C40:3	0.054	-0.118	2.185	0.007
PC ae C40:6	0.051	-0.106	2.078	0.007
PC ae C44:4	0.017	-0.080	4.706	0.011
PC aa C28:1	0.121	-0.581	4.802	0.014
PC aa C42:0	0.041	-0.088	2.146	0.014
Hex2Cer(d18:1/16:0)	0.557	-3.112	5.587	<0.001
Hex2Cer(d18:1/24:1)	0.181	-0.910	5.028	<0.001
HexCer(d18:1/22:0)	0.063	-0.971	15.413	<0.001
HexCer(d18:1/20:0)	0.063	-0.203	3.222	<0.001
Hex3Cer(d18:1/16:0)	0.458	-1.032	2.253	<0.001
Hex2Cer(d18:1/22:0)	0.038	-0.172	4.526	<0.001
HexCer(d18:1/24:0)	0.070	-0.302	4.314	0.001
Hex3Cer(d18:1/24:1)	0.086	-0.823	9.570	0.001
HexCer(d18:1/16:0)	0.386	-0.958	2.482	0.002
HexCer(d18:1/24:1)	-0.122	-1.454	11.918	0.015
SM C16:1	0.606	-1.123	1.853	<0.001
SM (OH) C22:2	0.140	-0.237	1.693	<0.001
SM C18:1	0.259	-0.411	1.587	<0.001
SM (OH) C22:1	0.133	-0.680	5.113	<0.001
SM C24:0	0.644	-1.793	2.784	<0.001
SM (OH) C14:1	0.215	-0.400	1.860	<0.001
SM C24:1	1.406	-4.094	2.912	<0.001

SM C18:0	1.027	-1.555	1.514	0.002
SM C22:3	-0.003	-0.011	3.667	0.041
SM C16:0	3.160	-5.256	1.663	0.048
TG(18:1_36:3)	1.448	-3.257	2.249	<0.001
TG(18:2_36:2)	0.763	-1.450	1.900	0.004
TG(18:1_36:4)	0.526	-0.996	1.894	0.004
TG(18:2_34:0)	0.164	-0.431	2.628	0.008
TG(18:1_34:2)	1.424	-3.364	2.362	0.012
TG(18:0_36:3)	0.197	-0.324	1.645	0.044

[a] Metabolite change after successful *H. pylori* eradication was calculated by the mean difference of metabolite level after successful eradication and baseline; [b] Metabolite change after failed treatment was calculated by the mean difference of metabolite level after failed treatment and baseline; [c] FC, fold change was calculated as the ratio of the mean difference of metabolite after successful eradication to the mean difference of metabolites after failed treatment; [d] Mann-Whitney U test with *P* values adjusted for multiple comparison by FDR.

Supplementary Table S3. Significant correlations between differential metabolites and gastric genera in baseline IM lesion

Differential Metabolites	Differential gastric genera	*r* [a]	*P* value [a]
CE(18:2)	*Staphylococcus*	-0.485	<0.001
CE(18:2)	*Corynebacterium*	-0.318	0.028
CE(20:4)	*Staphylococcus*	-0.428	0.002
PC aa C34:4	*Alloprevotella*	-0.425	0.003
PC aa C34:4	*Prevotella*	-0.395	0.005
PC aa C34:4	*Gemella*	-0.353	0.014
PC aa C34:4	*Streptococcus*	-0.344	0.017
PC aa C34:4	*Neisseria*	-0.341	0.018
PC aa C34:4	*Veillonella*	-0.341	0.018
PC aa C34:4	*Fusobacterium*	-0.339	0.018
PC aa C34:4	*Porphyromonas*	-0.330	0.022
PC aa C34:4	*Haemophilus*	-0.295	0.041
PC aa C34:4	*Helicobacter*	0.290	0.046
PC aa C42:5	*Alloprevotella*	-0.298	0.040
PC ae C36:4	*Staphylococcus*	-0.422	0.003
PC ae C38:5	*Staphylococcus*	-0.289	0.046
PC ae C44:5	*Staphylococcus*	-0.316	0.029
Hex2Cer(d18:1/16:0)	*Streptococcus*	-0.377	0.008
Hex2Cer(d18:1/16:0)	*Porphyromonas*	-0.364	0.011
Hex2Cer(d18:1/16:0)	*Haemophilus*	-0.364	0.011
Hex2Cer(d18:1/16:0)	*Gemella*	-0.356	0.013
Hex2Cer(d18:1/16:0)	*Fusobacterium*	-0.350	0.015
Hex2Cer(d18:1/16:0)	*Staphylococcus*	-0.339	0.018
Hex2Cer(d18:1/16:0)	*Corynebacterium*	-0.293	0.043
Hex2Cer(d18:1/16:0)	*Neisseria*	-0.286	0.049
Hex2Cer(d18:1/22:0)	*Porphyromonas*	-0.388	0.006
Hex2Cer(d18:1/22:0)	*Fusobacterium*	-0.354	0.014
Hex2Cer(d18:1/22:0)	*Gemella*	-0.353	0.014
Hex2Cer(d18:1/22:0)	*Streptococcus*	-0.328	0.023
Hex2Cer(d18:1/22:0)	*Haemophilus*	-0.304	0.036
Hex2Cer(d18:1/24:1)	*Porphyromonas*	-0.369	0.010
Hex2Cer(d18:1/24:1)	*Haemophilus*	-0.351	0.014
Hex2Cer(d18:1/24:1)	*Streptococcus*	-0.346	0.016
Hex2Cer(d18:1/24:1)	*Gemella*	-0.345	0.016

Hex2Cer(d18:1/24:1)	*Fusobacterium*	-0.328	0.023
Hex3Cer(d18:1/24:1)	*Corynebacterium*	-0.338	0.019
Hex3Cer(d18:1/24:1)	*Staphylococcus*	-0.322	0.026
SM C22:3	*Staphylococcus*	-0.331	0.022
TG(18:0_36:3)	*Prevotella*	0.291	0.045

[a] Spearman correlation analysis.

Supplementary Table S4. Significant correlations between differential metabolites and gastric genera in IM lesion after treatment

Differential Metabolites	Differential gastric genera	r [a]	P value [a]
Cer(d18:1/22:0)	*Helicobacter*	0.342	0.007
Cer(d18:1/22:0)	*Alloprevotella*	-0.286	0.024
Cer(d18:1/22:0)	*Corynebacterium*	-0.324	0.010
Cer(d18:1/22:0)	*Bacteroides*	-0.325	0.010
Cer(d18:1/24:0)	*Helicobacter*	0.393	0.002
Cer(d18:1/24:0)	*Prevotella*	-0.268	0.035
Cer(d18:1/24:0)	*Neisseria*	-0.293	0.021
Cer(d18:1/24:0)	*Staphylococcus*	-0.313	0.013
Cer(d18:1/24:0)	*Alloprevotella*	-0.328	0.009
Cer(d18:1/24:0)	*Fusobacterium*	-0.328	0.009
Cer(d18:1/24:0)	*Bacteroides*	-0.375	0.003
Cer(d18:1/24:0)	*Corynebacterium*	-0.384	0.002
Cer(d18:1/24:1)	*Helicobacter*	0.410	0.001
Cer(d18:1/24:1)	*Staphylococcus*	-0.264	0.038
Cer(d18:1/24:1)	*Neisseria*	-0.318	0.012
Cer(d18:1/24:1)	*Bacteroides*	-0.325	0.010
Cer(d18:1/24:1)	*Prevotella*	-0.327	0.010
Cer(d18:1/24:1)	*Porphyromonas*	-0.329	0.009
Cer(d18:1/24:1)	*Corynebacterium*	-0.350	0.005
Cer(d18:1/24:1)	*Fusobacterium*	-0.360	0.004
Cer(d18:1/24:1)	*Alloprevotella*	-0.375	0.003
CE(18:2)	*Helicobacter*	0.341	0.007
CE(18:2)	*Porphyromonas*	-0.258	0.043
CE(18:2)	*Rothia*	-0.288	0.023
CE(18:2)	*Prevotella*	-0.297	0.019
CE(18:2)	*Fusobacterium*	-0.305	0.016
CE(18:2)	*Gemella*	-0.349	0.005
CE(18:2)	*Streptococcus*	-0.352	0.005
CE(18:2)	*Corynebacterium*	-0.364	0.004
CE(18:2)	*Staphylococcus*	-0.378	0.002
CE(20:4)	*Helicobacter*	0.389	0.002
CE(20:4)	*Bacteroides*	-0.266	0.036
CE(20:4)	*Alloprevotella*	-0.274	0.031
CE(20:4)	*Streptococcus*	-0.291	0.022

CE(20:4)	*Gemella*	-0.294	0.021
CE(20:4)	*Staphylococcus*	-0.297	0.019
CE(20:4)	*Fusobacterium*	-0.316	0.012
CE(20:4)	*Prevotella*	-0.316	0.012
CE(20:4)	*Corynebacterium*	-0.358	0.004
FA(20:3)	*Helicobacter*	0.282	0.027
FA(20:3)	*Fusobacterium*	-0.271	0.033
lysoPC a C20:4	*Helicobacter*	0.402	0.001
lysoPC a C20:4	*Bacteroides*	-0.265	0.037
lysoPC a C20:4	*Porphyromonas*	-0.268	0.035
lysoPC a C20:4	*Neisseria*	-0.271	0.033
lysoPC a C20:4	*Rothia*	-0.272	0.033
lysoPC a C20:4	*Prevotella*	-0.290	0.022
lysoPC a C20:4	*Staphylococcus*	-0.362	0.004
lysoPC a C20:4	*Fusobacterium*	-0.376	0.003
lysoPC a C20:4	*Corynebacterium*	-0.463	<0.001
PC aa C32:0	*Helicobacter*	0.335	0.008
PC aa C32:0	*Staphylococcus*	-0.251	0.049
PC aa C32:0	*Corynebacterium*	-0.343	0.006
PC aa C36:4	*Helicobacter*	0.417	0.001
PC aa C36:4	*Rothia*	-0.253	0.048
PC aa C36:4	*Fusobacterium*	-0.308	0.015
PC aa C36:4	*Bacteroides*	-0.336	0.008
PC aa C36:4	*Staphylococcus*	-0.393	0.002
PC aa C36:4	*Corynebacterium*	-0.487	<0.001
PC aa C38:4	*Helicobacter*	0.311	0.014
PC aa C38:4	*Staphylococcus*	-0.262	0.040
PC aa C38:4	*Bacteroides*	-0.278	0.029
PC aa C38:4	*Corynebacterium*	-0.370	0.003
PC ae C32:1	*Helicobacter*	0.328	0.009
PC ae C32:1	*Bacteroides*	-0.303	0.017
PC ae C32:1	*Corynebacterium*	-0.323	0.011
PC ae C36:4	*Helicobacter*	0.360	0.004
PC ae C36:4	*Prevotella*	-0.270	0.034
PC ae C36:4	*Fusobacterium*	-0.291	0.022
PC ae C36:4	*Corynebacterium*	-0.348	0.006
PC ae C36:4	*Bacteroides*	-0.380	0.002
PC ae C36:5	*Helicobacter*	0.405	0.001

PC ae C36:5	*Fusobacterium*	-0.322	0.011
PC ae C36:5	*Staphylococcus*	-0.351	0.005
PC ae C36:5	*Bacteroides*	-0.367	0.003
PC ae C36:5	*Corynebacterium*	-0.475	<0.001
PC ae C38:4	*Helicobacter*	0.273	0.032
PC ae C38:4	*Bacteroides*	-0.266	0.037
PC ae C38:4	*Corynebacterium*	-0.314	0.013
PC ae C38:5	*Helicobacter*	0.335	0.008
PC ae C38:5	*Bacteroides*	-0.292	0.021
PC ae C38:5	*Corynebacterium*	-0.366	0.003
PC ae C38:6	*Helicobacter*	0.276	0.030
PC ae C44:5	*Streptococcus*	-0.262	0.040
Hex2Cer(d18:1/16:0)	*Helicobacter*	0.708	<0.001
Hex2Cer(d18:1/16:0)	*Bacteroides*	-0.416	0.001
Hex2Cer(d18:1/16:0)	*Staphylococcus*	-0.427	0.001
Hex2Cer(d18:1/16:0)	*Haemophilus*	-0.485	<0.001
Hex2Cer(d18:1/16:0)	*Veillonella*	-0.526	<0.001
Hex2Cer(d18:1/16:0)	*Neisseria*	-0.530	<0.001
Hex2Cer(d18:1/16:0)	*Corynebacterium*	-0.531	<0.001
Hex2Cer(d18:1/16:0)	*Porphyromonas*	-0.535	<0.001
Hex2Cer(d18:1/16:0)	*Alloprevotella*	-0.548	<0.001
Hex2Cer(d18:1/16:0)	*Streptococcus*	-0.555	<0.001
Hex2Cer(d18:1/16:0)	*Rothia*	-0.562	<0.001
Hex2Cer(d18:1/16:0)	*Prevotella*	-0.568	<0.001
Hex2Cer(d18:1/16:0)	*Gemella*	-0.582	<0.001
Hex2Cer(d18:1/16:0)	*Fusobacterium*	-0.627	<0.001
Hex2Cer(d18:1/22:0)	*Helicobacter*	0.473	<0.001
Hex2Cer(d18:1/22:0)	*Bacteroides*	-0.268	0.035
Hex2Cer(d18:1/22:0)	*Corynebacterium*	-0.285	0.025
Hex2Cer(d18:1/22:0)	*Veillonella*	-0.348	0.006
Hex2Cer(d18:1/22:0)	*Rothia*	-0.369	0.003
Hex2Cer(d18:1/22:0)	*Streptococcus*	-0.390	0.002
Hex2Cer(d18:1/22:0)	*Prevotella*	-0.395	0.001
Hex2Cer(d18:1/22:0)	*Alloprevotella*	-0.430	<0.001
Hex2Cer(d18:1/22:0)	*Gemella*	-0.435	<0.001
Hex2Cer(d18:1/22:0)	*Haemophilus*	-0.451	<0.001
Hex2Cer(d18:1/22:0)	*Porphyromonas*	-0.505	<0.001
Hex2Cer(d18:1/22:0)	*Neisseria*	-0.518	<0.001

Hex2Cer(d18:1/22:0)	*Fusobacterium*	-0.520	<0.001
Hex2Cer(d18:1/24:1)	*Helicobacter*	0.572	<0.001
Hex2Cer(d18:1/24:1)	*Haemophilus*	-0.383	0.002
Hex2Cer(d18:1/24:1)	*Neisseria*	-0.402	0.001
Hex2Cer(d18:1/24:1)	*Staphylococcus*	-0.404	0.001
Hex2Cer(d18:1/24:1)	*Veillonella*	-0.420	0.001
Hex2Cer(d18:1/24:1)	*Porphyromonas*	-0.431	<0.001
Hex2Cer(d18:1/24:1)	*Bacteroides*	-0.436	<0.001
Hex2Cer(d18:1/24:1)	*Rothia*	-0.454	<0.001
Hex2Cer(d18:1/24:1)	*Alloprevotella*	-0.461	<0.001
Hex2Cer(d18:1/24:1)	*Prevotella*	-0.465	<0.001
Hex2Cer(d18:1/24:1)	*Streptococcus*	-0.478	<0.001
Hex2Cer(d18:1/24:1)	*Gemella*	-0.501	<0.001
Hex2Cer(d18:1/24:1)	*Fusobacterium*	-0.502	<0.001
Hex2Cer(d18:1/24:1)	*Corynebacterium*	-0.516	<0.001
Hex3Cer(d18:1/16:0)	*Helicobacter*	0.255	0.045
Hex3Cer(d18:1/16:0)	*Bacteroides*	-0.258	0.043
Hex3Cer(d18:1/16:0)	*Corynebacterium*	-0.269	0.035
Hex3Cer(d18:1/24:1)	*Helicobacter*	0.280	0.028
Hex3Cer(d18:1/24:1)	*Fusobacterium*	-0.259	0.042
Hex3Cer(d18:1/24:1)	*Bacteroides*	-0.264	0.038
HexCer(d18:1/16:0)	*Helicobacter*	0.257	0.044
HexCer(d18:1/16:0)	*Fusobacterium*	-0.271	0.033
HexCer(d18:1/20:0)	*Helicobacter*	0.265	0.037
HexCer(d18:1/20:0)	*Alloprevotella*	-0.264	0.038
HexCer(d18:1/20:0)	*Haemophilus*	-0.290	0.022
HexCer(d18:1/20:0)	*Gemella*	-0.303	0.017
HexCer(d18:1/20:0)	*Fusobacterium*	-0.311	0.014
HexCer(d18:1/20:0)	*Prevotella*	-0.323	0.010
HexCer(d18:1/20:0)	*Neisseria*	-0.340	0.007
HexCer(d18:1/20:0)	*Porphyromonas*	-0.383	0.002
HexCer(d18:1/22:0)	*Helicobacter*	0.369	0.003
HexCer(d18:1/22:0)	*Neisseria*	-0.260	0.042
HexCer(d18:1/22:0)	*Bacteroides*	-0.264	0.038
HexCer(d18:1/22:0)	*Rothia*	-0.271	0.033
HexCer(d18:1/22:0)	*Porphyromonas*	-0.274	0.031
HexCer(d18:1/22:0)	*Alloprevotella*	-0.280	0.027
HexCer(d18:1/22:0)	*Haemophilus*	-0.291	0.022

HexCer(d18:1/22:0)	*Prevotella*	-0.304	0.016
HexCer(d18:1/22:0)	*Staphylococcus*	-0.305	0.016
HexCer(d18:1/22:0)	*Fusobacterium*	-0.318	0.012
HexCer(d18:1/22:0)	*Streptococcus*	-0.343	0.006
HexCer(d18:1/22:0)	*Gemella*	-0.344	0.006
HexCer(d18:1/22:0)	*Corynebacterium*	-0.375	0.003
HexCer(d18:1/24:0)	*Helicobacter*	0.301	0.017
HexCer(d18:1/24:0)	*Neisseria*	-0.258	0.043
HexCer(d18:1/24:0)	*Porphyromonas*	-0.263	0.039
HexCer(d18:1/24:0)	*Staphylococcus*	-0.265	0.038
HexCer(d18:1/24:0)	*Haemophilus*	-0.265	0.037
HexCer(d18:1/24:0)	*Fusobacterium*	-0.273	0.032
HexCer(d18:1/24:0)	*Prevotella*	-0.275	0.031
HexCer(d18:1/24:0)	*Streptococcus*	-0.276	0.030
HexCer(d18:1/24:0)	*Corynebacterium*	-0.302	0.017
HexCer(d18:1/24:1)	*Helicobacter*	0.287	0.024
HexCer(d18:1/24:1)	*Neisseria*	-0.255	0.045
HexCer(d18:1/24:1)	*Staphylococcus*	-0.257	0.043
HexCer(d18:1/24:1)	*Fusobacterium*	-0.258	0.043
HexCer(d18:1/24:1)	*Prevotella*	-0.260	0.041
HexCer(d18:1/24:1)	*Corynebacterium*	-0.286	0.024
HexCer(d18:1/24:1)	*Alloprevotella*	-0.339	0.007
SM (OH) C22:1	*Fusobacterium*	-0.257	0.044
SM C22:3	*Helicobacter*	0.289	0.023
SM C16:0	*Prevotella*	0.268	0.036
TG(18:0_36:3)	*Prevotella*	0.365	0.004
TG(18:0_36:3)	*Veillonella*	0.319	0.012
TG(18:0_36:3)	*Porphyromonas*	0.310	0.014
TG(18:0_36:3)	*Neisseria*	0.303	0.017
TG(18:0_36:3)	*Rothia*	0.302	0.017
TG(18:0_36:3)	*Fusobacterium*	0.276	0.030
TG(18:0_36:3)	*Alloprevotella*	0.259	0.042
TG(18:1_36:4)	*Helicobacter*	0.252	0.048
TG(18:2_36:2)	*Helicobacter*	-0.264	0.038

[a] Spearman correlation analysis.

Supplementary Table S5. Relationships between differential genera associated metabolites and IM lesions in baseline subjects [a]

Differential genera associated metabolites	SG/CAG (Mean ± SD)	IM (Mean ± SD)	B	Std. Error	*OR*	95% Wald Confidence Interval for *OR*		*P*-value
						Lower	Upper	
Cer(d18:1/22:0)	0.97 ± 0.42	1.19 ± 0.50	0.052	0.066	1.053	0.925	1.199	0.437
Cer(d18:1/24:0)	1.89 ± 0.65	1.63 ± 0.73	-0.104	0.579	0.901	0.290	2.801	0.857
Cer(d18:1/24:1)	4.03 ± 1.56	3.33 ± 1.41	-0.813	0.337	0.444	0.229	0.858	0.016
CE(18:2)	21.00 ± 16.08	21.67 ± 17.52	0.002	0.011	1.002	0.981	1.024	0.824
CE(20:4)	11.47 ± 7.01	11.50 ± 6.68	0.050	0.029	1.052	0.993	1.113	0.085
FA(20:3)	62.29 ± 40.43	75.62 ± 42.65	0.010	0.005	1.010	1.001	1.019	0.049
lysoPC a C20:4	2.92 ± 1.52	2.66 ± 1.57	-0.214	0.118	0.807	0.641	1.018	0.071
PC aa C32:0	47.71 ± 17.38	36.4 ± 15.19	-0.023	0.017	0.977	0.945	1.010	0.175
PC aa C34:4	0.10 ± 0.10	0.09 ± 0.08	-0.121	0.187	0.886	0.614	1.278	0.517
PC aa C36:4	20.01 ± 8.08	22.20 ± 8.12	0.043	0.035	1.044	0.975	1.118	0.221
PC aa C38:4	13.62 ± 5.16	12.42 ± 5.45	-0.016	0.061	0.984	0.873	1.109	0.794
PC aa C42:5	0.17 ± 0.12	0.21 ± 0.16	0.039	0.103	1.040	0.850	1.272	0.707
PC ae C32:1	4.74 ± 2.29	5.60 ± 1.98	0.323	0.165	1.381	1.001	1.909	0.049
PC ae C36:4	3.21 ± 1.39	3.00 ± 1.53	-0.081	0.098	0.922	0.761	1.117	0.408
PC ae C36:5	2.06 ± 0.96	1.92 ± 1.10	-0.312	0.315	0.732	0.395	1.357	0.323
PC ae C38:4	1.74 ± 0.94	1.81 ± 0.80	0.105	0.344	1.111	0.566	2.180	0.760
PC ae C38:5	2.30 ± 1.31	2.44 ± 1.08	0.270	0.406	1.310	0.591	2.903	0.506
PC ae C38:6	1.23 ± 0.61	1.19 ± 0.63	-0.494	0.337	0.610	0.315	1.181	0.143
PC ae C44:5	0.31 ± 0.19	0.30 ± 0.15	-0.008	0.087	0.992	0.837	1.176	0.930
Hex2Cer(d18:1/16:0)	5.33 ± 2.93	4.45 ± 3.33	-0.051	0.150	0.950	0.708	1.275	0.732
Hex2Cer(d18:1/22:0)	0.57 ± 0.29	0.51 ± 0.35	-0.130	0.731	0.878	0.210	3.679	0.859
Hex2Cer(d18:1/24:1)	1.51 ± 1.14	1.76 ± 0.96	0.137	0.526	1.147	0.409	3.215	0.795
Hex3Cer(d18:1/16:0)	4.31 ± 1.78	3.52 ± 1.72	-0.077	0.176	0.926	0.656	1.307	0.661
Hex3Cer(d18:1/24:1)	1.99 ± 0.98	2.55 ± 1.29	0.313	0.248	1.368	0.841	2.223	0.207
HexCer(d18:1/16:0)	4.85 ± 2.02	3.95 ± 2.10	-0.230	0.129	0.794	0.617	1.023	0.074
HexCer(d18:1/20:0)	0.53 ± 0.31	0.36 ± 0.31	-0.254	0.517	0.776	0.282	2.137	0.624
HexCer(d18:1/22:0)	3.00 ± 1.31	2.13 ± 1.23	-0.276	0.203	0.759	0.510	1.130	0.175
HexCer(d18:1/24:0)	1.34 ± 0.50	1.04 ± 0.55	-0.496	0.462	0.609	0.246	1.506	0.284
HexCer(d18:1/24:1)	8.20 ± 2.83	8.86 ± 2.58	0.206	0.085	1.229	1.040	1.452	0.016
SM (OH) C22:1	1.69 ± 1.03	2.75 ± 0.99	0.784	0.283	2.191	1.258	3.814	0.006
SM C16:0	124.09 ± 43.72	102.36 ± 41.30	-0.003	0.003	0.997	0.991	1.003	0.353
SM C22:3	0.04 ± 0.06	0.03 ± 0.05	-0.140	0.162	0.870	0.633	1.194	0.389

TG(18:0_36:3)	0.87 ± 1.17	0.53 ± 0.76	-0.338	0.169	0.713	0.512	0.993	0.045
TG(18:1_36:4)	2.64 ± 3.83	2.16 ± 4.11	-0.048	0.045	0.953	0.873	1.041	0.288
TG(18:2_36:2)	2.81 ± 2.89	3.07 ± 3.33	0.075	0.039	1.078	0.998	1.164	0.058

[a] Unconditional logistic regression adjusted for age and gender.

Supplementary Table S6. Relationships between differential genera associated metabolites and IM lesion in follow-up subjects after anti-*H. pylori* treatment [a]

Gastric genera associated metabolites	SG/CAG (Mean ± SD)	IM (Mean ± SD)	B	Std. Error	*OR*	95% Wald Confidence Interval for *OR*		*P*-value
						Lower	Upper	
Cer(d18:1/22:0)	1.01 ± 0.33	0.94 ± 0.56	-3.753	1.767	0.023	0.001	0.749	0.034
Cer(d18:1/24:0)	1.58 ± 0.91	1.60 ± 0.46	0.238	0.127	1.269	0.989	1.627	0.061
Cer(d18:1/24:1)	3.43 ± 1.10	3.29 ± 1.77	-0.334	0.366	0.716	0.350	1.467	0.361
CE(18:2)	18.56 ± 13.29	18.29 ± 13.88	-0.014	0.011	0.986	0.965	1.008	0.190
CE(20:4)	10.00 ± 5.26	10.02 ± 8.86	0.006	0.017	1.006	0.973	1.040	0.739
FA(20:3)	53.15 ± 27.01	55.71 ± 38.80	0.0004	0.003	1.000	0.995	1.006	0.876
lysoPC a C20:4	2.13 ± 1.63	2.14 ± 1.33	0.088	0.107	1.092	0.885	1.347	0.411
PC aa C32:0	34.04 ± 8.48	34.01 ± 14.58	0.027	0.026	1.027	0.976	1.081	0.304
PC aa C34:4	0.06 ± 0.07	0.05 ± 0.05	-0.117	0.215	0.890	0.584	1.356	0.587
PC aa C36:4	16.38 ± 8.68	16.20 ± 7.81	-0.126	0.074	0.882	0.763	1.019	0.089
PC aa C38:4	9.87 ± 4.99	10.22 ± 5.56	0.154	0.127	1.166	0.910	1.496	0.225
PC aa C42:5	0.17 ± 0.15	0.16 ± 0.13	-0.047	0.026	0.954	0.907	1.004	0.069
PC ae C32:1	4.37 ± 1.62	4.37 ± 2.23	-0.019	0.170	0.981	0.703	1.369	0.912
PC ae C36:4	2.53 ± 1.40	2.67 ± 1.25	0.086	0.050	1.090	0.988	1.202	0.087
PC ae C36:5	1.37 ± 0.85	1.25 ± 0.73	-0.251	0.421	0.778	0.341	1.776	0.550
PC ae C38:4	1.30 ± 0.72	1.36 ± 0.77	0.193	0.122	1.213	0.955	1.541	0.114
PC ae C38:5	1.67 ± 1.02	1.64 ± 0.96	-1.864	1.068	0.155	0.019	1.258	0.081
PC ae C38:6	0.77 ± 0.48	0.84 ± 0.61	0.028	0.015	1.028	0.999	1.059	0.056
PC ae C44:5	0.26 ± 0.17	0.23 ± 0.14	-3.119	2.193	0.044	0.001	3.252	0.155
Hex2Cer(d18:1/16:0)	3.49 ± 3.82	3.03 ± 2.95	-0.096	0.158	0.908	0.667	1.238	0.546
Hex2Cer(d18:1/22:0)	0.39 ± 0.33	0.43 ± 0.31	0.004	0.002	1.004	1.001	1.008	0.032
Hex2Cer(d18:1/24:1)	1.21 ± 1.14	1.04 ± 0.95	-0.141	0.611	0.868	0.262	2.876	0.817
Hex3Cer(d18:1/16:0)	3.54 ± 1.07	3.01 ± 1.53	-0.832	0.362	0.435	0.214	0.885	0.022
Hex3Cer(d18:1/24:1)	1.90 ± 0.94	2.06 ± 0.66	0.479	0.319	1.614	0.864	3.017	0.133
HexCer(d18:1/16:0)	4.30 ± 1.44	3.40 ± 2.06	-0.774	0.427	0.461	0.200	1.065	0.070
HexCer(d18:1/20:0)	0.42 ± 0.35	0.32 ± 0.29	-0.286	0.299	0.751	0.418	1.350	0.338
HexCer(d18:1/22:0)	2.02 ± 1.35	2.18 ± 1.16	0.193	0.149	1.213	0.906	1.624	0.195
HexCer(d18:1/24:0)	0.98 ± 0.52	1.13 ± 0.39	0.547	0.401	1.728	0.787	3.792	0.172
HexCer(d18:1/24:1)	8.44 ± 2.20	7.79 ± 3.19	-0.005	0.067	0.995	0.873	1.135	0.940
SM (OH) C22:1	2.38 ± 0.85	2.47 ± 1.12	0.067	0.042	1.069	0.985	1.161	0.112
SM C16:0	107.07 ± 42.2	110.96 ± 24.13	0.015	0.009	1.015	0.997	1.033	0.084
SM C22:3	0.04 ± 0.05	0.03 ± 0.05	-0.104	0.056	0.901	0.808	1.006	0.066

TG(18:0_36:3)	0.61 ± 0.81	0.33 ± 0.56	-0.581	0.427	0.559	0.242	1.292	0.174
TG(18:1_36:4)	1.57 ± 3.52	1.37 ± 3.19	-0.067	0.053	0.935	0.843	1.038	0.203
TG(18:2_36:2)	2.42 ± 2.29	1.62 ± 1.80	-0.309	0.158	0.734	0.539	1.001	0.051

[a] Unconditional logistic regression adjusted for age, gender and effect of interventions.
